# Uncoupling of the dynamics of host–pathogen interaction uncovers new mechanisms of viral interferon antagonism at the single-cell level

**DOI:** 10.1093/nar/gku492

**Published:** 2014-06-04

**Authors:** Ulfert Rand, Upneet Hillebrand, Stephanie Sievers, Steffi Willenberg, Mario Köster, Hansjörg Hauser, Dagmar Wirth

**Affiliations:** 1 Model Systems for Infection and Immunity, Helmholtz Centre for Infection Research, Inhoffenstrasse 7, 38124 Braunschweig, Germany; 2 Gene Regulation and Differentiation, Helmholtz Centre for Infection Research, Inhoffenstrasse 7, 38124 Braunschweig, Germany

## Abstract

Antiviral defence in mammals is mediated through type-I interferons (IFNs). Viruses antagonise this process through expression of IFN antagonist proteins (IAPs). Understanding and modelling of viral escape mechanisms and the dynamics of IAP action has the potential to facilitate the development of specific and safe drugs. Here, we describe the dynamics of interference by selected viral IAPs, NS1 from Influenza A virus and NS3/4A from Hepatitis C virus. We used Tet-inducible IAP gene expression to uncouple this process from virus-driven dynamics. Stochastic activation of the IFN-β gene required the use of single-cell live imaging to define the efficacy of the inhibitors during the virus-induced signalling processes. We found significant correlation between the onset of IAP expression and halted IFN-β expression in cells where IFN-β induction had already occurred. These data indicate that IAPs not only prevent antiviral signalling prior to IFN-β induction, but can also stop the antiviral response even after it has been activated. We found reduced NF-κB activation to be the underlying mechanism by which activated IFN expression can be blocked. This work demonstrates a new mechanism by which viruses can antagonise the IFN response.

## INTRODUCTION

The cellular recognition of pathogen-derived nucleic acids evokes early cellular defence mechanisms like the secretion of type-I interferons (IFNs). The antiviral IFN response is raised from discrete infected cells (1) and elicits protection through paracrine and autocrine stimulation (2–5). The cascade of molecular events following infection has been extensively studied. One mechanism makes use of binding of viral nucleic acids to cellular pathogen recognition receptors (PRRs) such as MDA5 and RIG-I, leading to their subsequent activation. This initiates downstream signalling via the mitochondrial protein MAVS and its associated complex. The kinases TBK-1/IKK-ϵ activate IRF-3 and IRF-7 leading to their homo- and heterodimerization and subsequent nuclear import. Simultaneously (and also initiated by MAVS), the nuclear accumulation of the main NF-κB complex, p50/p65, was found to be a consequence of IKKα/β/γ-mediated phosphorylation of IκBα and its degradation. Assembly of NF-κB, IRF-3/7 and AP-1 at the *Ifnb* promoter then initiates transcription. Importantly, there is substantial stochastic cell-to-cell variability in the timing of these activation processes. As a result, the onset of IFN-β expression varies from cell to cell (6).

Pathogenic viruses have evolved a plethora of functionally diverse interferon antagonist proteins (IAPs) to evade host immunity. IAPs often carry out more than one function combining different host immune evasion strategies with other roles in the virus life-cycle. Many of the immune response antagonising strategies target cellular signalling that leads to the induction of type-I IFN or its downstream effects (reviewed in (7)). This has a major impact on both viral spread and host survival. Action of the IAP NS1 from Influenza A virus (IAV) is considered a major target for antiviral treatment restoring the immune response (8). IAV NS1 (among other functions) blocks the activation of RIG-I via the TRIM25 ubiquitinating factor and this represents one of the earliest antagonistic targets of the IFN circuit. Apart from the Influenza virus, several (−)ssRNA viruses were found to mediate antagonism of the cellular RNA sensors, such as Ebola Virus, some Arenaviruses, Respiratory Syncytial Virus and multiple Paramyxoviridae. Hepatitis C virus protease complex NS3/NS4A was found to cleave MAVS, representing a novel strategy for immune evasion (9).

These mechanisms lead to a competition between activation of innate immune responses and viral antagonism. The dynamics of these processes are critically important for the functional outcome. The current understanding of virus-activated innate immune responses is mainly based on models where it is hypothesised that IFN activation evades viral antagonism, i.e. viral nucleic acid is sensed prior to presence of a functional antagonistic viral protein. This can be due to (i) the delay between amplification and translation of viral RNA and protein maturation, (ii) inhibition of viral protein translation through interferon-stimulated gene (ISG) products (10) and/or (iii) co-infection with virus particles failing to replicate or to antagonise RIG-I signalling.

We aimed to investigate the quantitative dynamics of these host–pathogen interactions by uncoupling expression of the viral antagonist from the virus life cycle. This was achieved by (i) the controlled expression of the viral inhibitors in cultured cells and (ii) the employment of Newcastle Disease Virus (NDV) as a model virus activating the IFN response through RIG-I (11,12), without antagonising this pathway. Alternatively, we used a synthetic dsRNA, low molecular weight polyinosinic:polycytidylic acid (LMW poly I:C), as a RIG-I ligand. In contrast to NDV RNA, Poly I:C is not replicated in the cell and thus allows a more controlled RIG-I stimulation. Previously developed IFN-β-TurboGFP reporter cells in which the TurboGFP reporter is integrated into the IFN-β locus within a bacterial artificial chromosome vector were used (6). These cells allow monitoring of IFN-β promoter activation by detection of the fast-folding bright fluorescent reporter TurboGFP in single cells. TurboGFP provides a fast and stable signal for activity of the *Ifnb* promoter while a halt of expression can be detected as a peak or plateaus. This has the advantage of not occupying other fluorescent spectra like fluorescence timer proteins. Further, in contrast to weak signals accompanied by many destabilized fluorescence proteins TurboGFP expression can be reliably detected by microscopy. IFN-β-TurboGFP cells revealed a considerable temporal cell-to-cell variation of IFN-β expression and its underlying IRF-3/7 and NF-κB activation (6). We stably integrated Tet-driven expression systems for viral antagonists into IFN-β-TurboGFP reporter cells. Viral antagonists were coupled to an affinity tag, allowing live-cell detection of its expression. Unexpectedly, we found that the doxycycline-induced Tet-on system also shows a considerable degree of temporal heterogeneity, requiring the use of single-cell analysis. Tracking both expression of the viral antagonist and expression of IFN-β revealed that the IFN-induction can be abrogated at any time point by the antagonist. Our data highlight the benefit of single cell imaging analysis for analysing transcriptionally regulated systems with intrinsic stochasticity.

## MATERIALS AND METHODS

### Plasmid construction

The coding sequence for IAV NS1 was amplified from pNS1.S8.SS2.NIBSC (kindly provided by Timo Frensing, Max-Planck Institute Magdeburg, Germany) using primers

NS1 5′: 5′-ttaatttcgatcgacgcgtccATGGATCCAAACACTGTGTCAAGC-3′ and

NS1 3′: 5′-gccgctagtcgcgaagatctgccaAACTTCTGACCTAATTGTTCC-3′ and ligated into pCMV.luc2.HTV7 (kindly provided by G. Los, Promega) using XhoII and PvuI restriction sites. Polymerase chain reaction (PCR) primer sequences were chosen to exclude the 3′ sequence of the nuclear export protein (NEP) splice variant. NS1-HaloTag fusion sequence (NS1-H) was excised with MluI/NotI and fused to the Tet-dependent promoter derived from pBI-1 (13) to generate ptet.NS1-H.

HCV NS3/4A coding sequence was amplified by PCR from pFK_i389lucEI.JFH1 dGDD (kind gift of Thomas Pietschmann, TWINCORE Hannover, Germany) using primers

NS3/4A_5′: 5′- ttaatttcgatcgacgcgtccatgGCTCCCATCACTGCTTATGC-3′ and

NS3/4A_3′: 5′-ttaatttgcggccgctagtcgcgaggatcccgCATTCCTCCATCTCATCAAAAGC-3′.

The PCR fragment was ligated into pCR-Blunt (Invitrogen) and excised via MluI and BamHI for subsequent sticky end ligation into MluI/BamHI opened ptet.NS1-H.

In pKrab.PGK.puro, the Cytomegalovirus (CMV) promoter drives the chimeric transrepressor tetR-KRAB consisting of the N-terminus of the KRAB repressor domain of the mammalian Kox1 protein fused to TetR (14). prtTA2.PGK.puro is the corresponding plasmid expressing the tetracycline-dependent reverse transactivator rtTA2^s^-M2 (15). Sequences and maps are available upon request.

### Cell culture, transfection, clone selection and fluorescent labelling

The murine fibroblast cell line NIH3T3 was cultured in Dulbecco's modified Eagle's medium (DMEM) supplemented with 10% foetal calf serum, penicillin, streptomycin and different combinations of antibiotics to select for stable vector integration (G418 [1 mg/ml], puromycin [2.5 μg/ml], hygromycin [300 U/ml]). NIH3T3 IFN-β-tGFP cells, described elsewhere (6), were transfected with either ptet.NS1-H or ptet-NS3/4A-H and the rtTA-encoding plasmid prtTA2.PGK.puro and the KRAB-transrepressor encoding plasmid pKrab.PGK.puro by lipofection using Metafectene (Biontex Laboratories) according to the manufacturer's instructions. Transfected cells were seeded at low density onto 10-cm dishes and clonal colonies were isolated. Cell clones were characterised for doxycycline-induced transgene expression and IFN-β-tGFP reporter expression. Representative clones exhibiting good inducibility upon addition of doxycycline and physiological IFN-β expression levels in the absence of doxycycline were used for further experiments. Cell lines were termed IFN-β-tGFP tet.NS1-H and IFN-β-tGFP tet.NS3/4A-H, respectively.

Fluorescent labelling of HaloTag-linked proteins was done by adding 300 nM HaloTag-ligand coupled TMR (Promega) for 30 min (for flow cytometric analysis) or supplementing medium with 20 nM HaloTag-ligand coupled TMR continuously for live-cell imaging.

### Flow cytometric analysis

Flow cytometry was performed using FACScalibur and LSR II (Becton Dickinson) equipped with 488 and 543 nm laser sources. Data analysis was performed with FlowJo software (Treestar).

### Virus infection and poly (I:C) stimulation

Infections with Newcastle Disease Virus strain LaSota (Lohmann Tierzucht, Cuxhaven, Germany) were performed after three washing steps with serum-free medium followed by 1 h incubation at 37°C and 5% CO_2_. Unattached virus particles in the supernatant were removed by washing three times with serum-containing medium. Low molecular weight poly (I:C) (InvivoGen) at 5 μg/ml final concentration was transfected via Lipofectamine 2000 (Invitrogen) according to the manufacturer's instructions.

### Recombinant IFN source and detection of IFN activity

Mouse IFN-β was produced in stably expressing BHK-21 cells and obtained from supernatant as described earlier (16). IFN activity was measured using a bioassay based on Mx2-Luc reporter gene expression (17).

### Immunofluorescence and confocal analysis

Cells were seeded on glass coverslips placed in 6-well plates (1.5–2 × 10^5^/well). Low molecular weight poly (I:C) (InvivoGen) at 5 μg/ml final concentration was transfected using Lipofectamine 2000 (Invitrogen) according to the manufacturer's instructions. Doxycycline (0.5 μg/ml) and TMR (100 nM) was added 2.15 h prior to poly (I:C) transfection. At the required time-points, cells were rinsed with phosphate buffered saline (PBS) and fixed with 4% paraformaldehyde for 10 min at room temperature followed by permeabilization with 0.1% Triton X-100 for 10 min at room temperature. Incubation with primary rabbit NF-κB p65 antibody (1:100) (Cell Signalling Technology, Catalogue No. 8242) for 1 h at room temperature was followed by washing three times with 3% bovine serum albumin (BSA). Fluorescein isothiocyanate (FITC)-labelled anti-rabbit secondary antibody (1:200) (Dianova, Catalogue No. 111-095-045) was added for 1 h at room temperature. Coverslips were mounted on glass slides using Fluoroshield mounting medium with DAPI (Sigma Aldrich) prior to microscopic analysis.

Confocal analysis was performed with a Zeiss LSM 510 META inverted confocal laser scanning microscope using a Plan NeoFluar 100× oil immersion objective (1.3 numeric aperture).

### Live-cell microscopy and image analysis

An Olympus IX81 fluorescence microscope equipped with a PRIOR motorized stage and climate chamber was used to acquire live-cell data from cells cultured in eight-well μ-slides coated with collagen IV (ibidi). The Olympus Cell^M software autofocus function was used to compensate Z-drift.

Image analysis was done using ImageJ (18) and plugins. Fluorescence profiles of single cells were generated using MTrackJ plugin (19).

## RESULTS

### Generation of double-labelled reporter cells to follow co-dynamics of IFN-β and viral antagonist action

**Figure 1.**
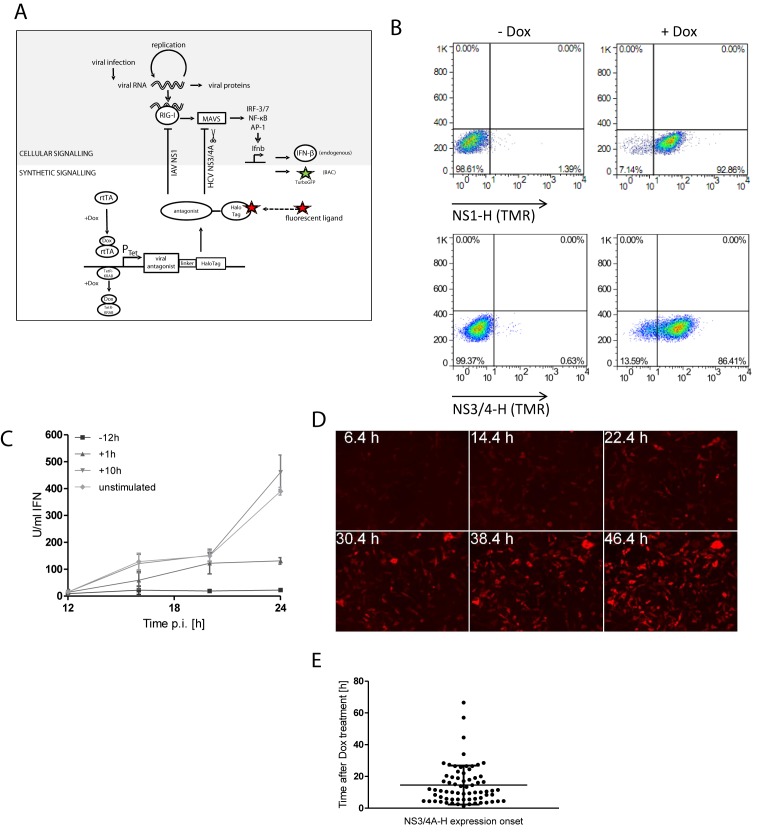
Cell lines reporting endogenous IFN signalling and synthetically expressing viral IAPs. (**A**) Schematic representation of endogenous and synthetic signalling. Virus infection and recognition of viral RNA by the intracellular receptor RIG-I lead to activation of transcription factors NF-κB, IRF-3/IRF-7 and AP-1 via the essential adaptor MAVS. The transcription factors activate the *Ifnb* promoter which controls the endogenous *Ifnb* gene as well as the BAC-encoded reporter TurboGFP. Tet-controlled expression of viral IAPs IAV NS1 or HCV NS3/4A each fused to HaloTag ligand binding domain is achieved via the reverse transactivator rtTA. The tet-repressor-KRAB fusion protein (TetR-KRAB) was implemented to eliminate basal expression in the absence of doxycyline. (**B**) Doxycycline-induced expression of Influenza A NS1 and HCV NS3/4A was detected by visualization of the HaloTag ligand with fluorescent TMR via flow cytometry at 48 h after addition of doxycycline. Untreated cells were used as controls. (**C**) Titres of type I IFN secreted from NDV infected NIH3T3 IFN-β-tGFP tet.NS1 cells upon addition of doxycycline (2 μg/ml) 12 h before infection with NDV as well as 1 and 10 h after infection were measured using Mx2-Luc bioassay published earlier (17). Titres from NDV infected cells without doxycycline treatment are shown as controls. (**D**) Time-lapse microscopy was employed to follow expression in individual cells over time (representative time frames are shown). (**E**) Cell-to-cell heterogeneity in the timing of doxycycline-induced NS1-expression as observed in time-lapse microscopy. Dots represent earliest detectable TMR fluorescence of individual cells (*n* = 69); 300 ms exposure; 20 nM TMR.

We chose IAV NS1 and HCV NS3/4A as two examples of viral proteins IAPs that interfere with RIG-I and MAVS signalling and originate from viruses with major clinical relevance. To direct the expression of these IAPs temporally and quantitatively, a doxycycline-dependent synthetic module was developed. NS1 and NS3/4A were fused to HaloTag which binds fluorescently labelled cell permeable ligands that can be quantified in living cells. To follow the consequences of specific perturbation of RIG-I/MAVS pathway on *Ifnb* promoter induction the synthetic expression modules were expressed in NIH3T3 IFN-β-tGFP reporter cells. In these cells TurboGFP (tGFP) is controlled by the IFN-β promoter in its native environment provided by a bacterial artificial chromosome (BAC) vector while they express physiological levels of IFN-β (6). Relevant endogenous signalling and the synthetic signalling modules are depicted in Figure 1A. By using the doxycycline-activated reverse transcriptional transactivator rtTA2^s^-M2 (13), we achieved to establish cell clones showing efficient induction of NS1 or NS3/4A and very low expression in absence of doxycycline (Figure 1B). Basal expression was suppressed with the doxycycline-responsive transrepressor TetR-KRAB (14). Cell clones were selected for lowest basal expression. Resulting inducible protein levels of NS1 induced in this system correspond to those in early IAV infection (6–12 h) in cell culture at moderate multiplicities of infection (MOI) (Supplementary Figure S1). NS1 showed dynamic nucleocytoplasmic movement in some cells (cf. Figure 3D), indicating that the HaloTag fusion did not interfere with function.

**Figure 2.**
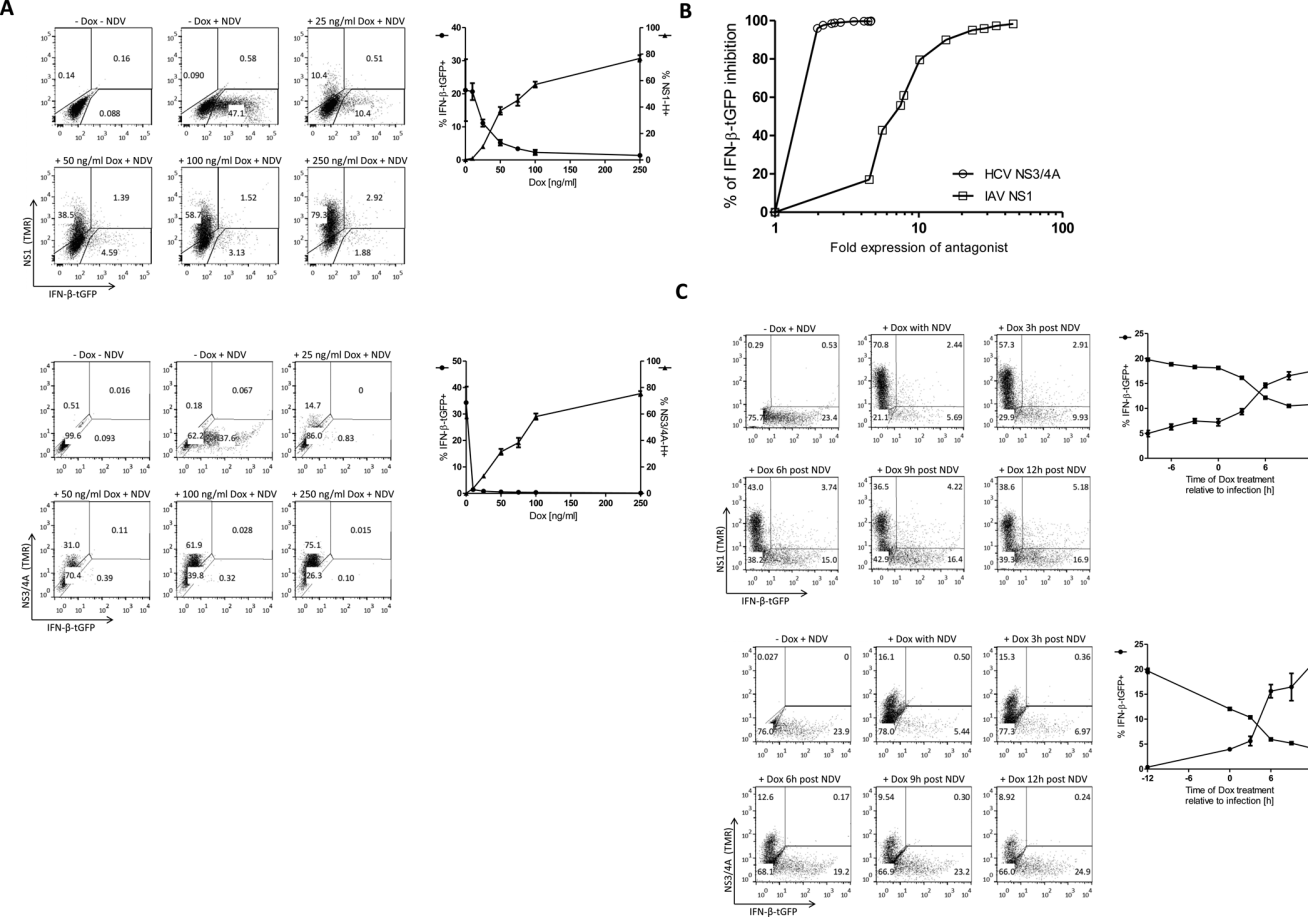
IAV NS1 and HCV NS3/4A display distinct time and concentration-dependent effects on type-I IFN expression. (**A**) tet.NS1 or tet.NS3/4A cells were treated with doxycycline concentrations (25–250 ng/ml) for 48 h and subsequently infected with NDV for 1 h. Thereby, the IAP is expressed in the majority of cells at the time of infection (cf. Figure 2E). Expression was determined 24 h after infection by flow cytometry. Dot plots are depicted in left panel, quantification of cell frequencies is given in right panel. (**B**) The relative inhibition of IFN-β-tGFP expression by increasing fold expression of the viral antagonist NS3/4A (open circles) and NS1 (open squares) was determined following increasing doses of doxycycline (10–2000 ng/ml). (**C**) tet.NS1 or tet.NS3/4A cells were treated with 2 μg/ml doxycycline prior to or post infection with NDV as indicated. Expression was determined 24 h after infection by flow cytometry. Dot plots are depicted in left panel, quantification of cell frequencies is given in right panel.

**Figure 3.**
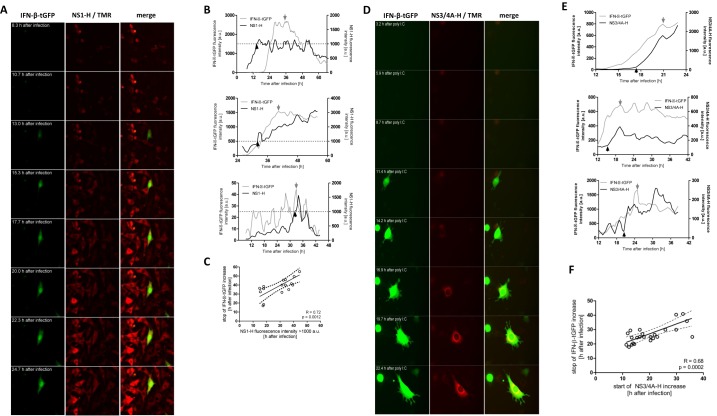
Effects of IAP expression after induction of IFN-β in single cells. Tet.NS1/IFN-β-tGFP and tet.NS3/4A/IFN-β-tGFP cells were infected with NDV for 1 h or transfected with poly I:C and stimulated with doxycycline 10 h later. Expression was followed by time-lapse microscopy. Depicted are representative images of cells expressing IFN-β-tGFP and NS-1 (**A**) as well as IFN-β-tGFP and NS3/4A (**D**). Cells that show both IFN-β-tGFP expression as well as IAP expression were tracked. Representative fluorescence intensity courses of three representative Tet.NS1/IFN-β-tGFP cells (**B**) and three representative tet.NS3/4A/ IFN-β-tGFP cells (**E**) are shown. (**C**) The time point when NS1 expression exceeded 1000 a.u. was plotted against time of maximal IFN-β-tGFP signal of the same cell. (**F**) Onset of fluorescent signal from NS3/4A-H/NS-1 bound TMR was plotted against time of IFN-β-tGFP peak signal where no further increase was observed. Each dot represents one cell, linear regression (straight line) and 95% confidence interval (dashed line). Significant correlation was concluded from the *P*-value calculated by *F*-test.

When doxycycline was applied to induce NS1 expression 12 h prior to Newcastle Disease Virus (NDV) infection a very low level of IFN activity was detectable in the supernatant (<10 U/ml; Figure 1C) at 12–24 h after infection. Thus, within this time frame efficient inhibition of IFN expression was achieved. Induction of NS1 1 h after infection repressed levels of secreted IFN strongly (<150 U/ml). This indicated that NS1 was able to repress RIG-I-mediated IFN-β induction after establishment of the virus. Late treatment with doxycycline (10 h postinfection) did not cause any significant effect over the untreated control (Figure 1C). Of note, this assay lacks the ability to track production of IFN-β in individual cells.

Monitoring the timing of Tet-induced expression by microscopic live-cell imaging, we unexpectedly found high cell-to-cell variation indicated by appearance of TMR fluorescence within individual cells (Figure 1D). This was observed in the isogenic cells at saturating concentrations of doxycycline excluding genetic variation and limiting conditions. Time of induction varied between 1.4 and 66.5 h after doxycycline administration (Figure 1D and E). This cell-to-cell heterogeneity of doxycycline-dependent gene induction was confirmed in several cell clones that were isolated independently from different transfections (data not shown). Strong temporal cell-to-cell heterogeneity was also observed in cells expressing a doxycycline-inducible GFP without control by a tetR-KRAB transrepressor, showing similar coefficients of variation (data not shown). Thus, doxycycline-driven activation of transgene expression underlies inherent cell-to-cell variation on a large temporal scale. Temporal variability is inherent to biological signalling systems and can range from seconds to days. For the induction of IFN-β expression we found that temporal variation could make cell-to-cell differences of more than 24 h (6). This probabilistic behaviour of both endogenous IFN-β induction and the Tet-induced antagonist expression limits the significance of population averaging assays. Thus, single-cell studies are necessary to analyse this host–pathogen interaction dynamically.

### IAV NS1 and HCV NS3/4A exhibit distinct dose- and time-dependent profiles of IFN-β inhibition

The temporal and quantitative mechanisms of action in the infected host cell are key properties of viral factors antagonising the host innate immune response. To compare these between IAV NS1 and NS3/4A, we measured the intensity and percentage of IFN-β expression upon infection with NDV over time and upon increasing amounts of doxycycline in our reporter system. Of note, NDV does not antagonise RIG-I signalling in mammalian cells (11,12).

To specifically evaluate the effects of IAV NS1 and HCV NS3/4A on IFN-β expression frequency within the infected cell population, we measured IFN-β-tGFP reporter expression by flow cytometry. As NS1 and NS3/4A are detected via the same fluorescent HaloTag-ligand given at saturating concentrations, relative expression levels and effective inhibition of IFN-β-tGFP expression could be compared in this system. In absence of doxycycline, the frequency of IFN-β expression was found to be comparable to cells not harbouring the antagonist expression constructs (Supplementary Figure S2). These data showed that the unstimulated expression from the synthetic IAP circuits did not significantly influence the system. Increasing concentrations of doxycycline led to a gradual decrease in the percentage of IFN-β-tGFP expression and reciprocally increasing numbers of NS1-expressing cells (Figure 2A, upper right panel). Interestingly, the reduction of IFN-β expressing cell counts was stronger than to be expected by the increase of cells gated for NS1-expression, indicating functional inhibition even below the detection limit of HaloTag-bound fluorescent ligand by flow cytometry.

Inhibition of IFN-β expression by NS3/4A under increasing doxycycline concentrations exhibited maximal effectiveness at much lower induction levels than compared to NS1. That was reflected in the sharp decline in its dose-response curve (Figure 2A, lower right panel). We suggest that this is due to its catalytic function (i.e. MAVS cleavage), while NS1 mediates a gradual inhibition of IFN-β activation.

This switch-like inhibition by NS3/4A is also observed in the time course of NS3/4A expression at saturating doxycycline levels. A sharp increase of IFN-expressing cells upon treatment between 3 and 6 h after infection was seen (Figure 2C) which correlated with the timing of IAP expression (cf. Figure 1D).

In summary, we observed different modes of antagonistic action for the two perturbators NS1 and NS3/4A. For maximal inhibition of virus-induced IFN-β-tGFP expression 4-fold induction of NS3/4A (over unstimulated levels) was needed. In contrast, NS1 levels needed to be raised more than 40-fold for maximal inhibition (Figure 2B, half-maximal inhibition at ∼1.5-fold (NS3/4A) or ∼6-fold (NS1) induction).

Our observations mirror the difference between the mechanisms of these viral antagonists: NS3/4A acts through a catalytic cleavage of MAVS (9), while NS-1 blocks by binding TRIM25 ubiquitin ligase thus preventing RIG-I activation (20). In summary, distinct characteristics and efficiency of viral IFN antagonism strategies can be detected with the experimental system described here.

### Delayed IAP induction correlates with halted IFN-β expression

Flow cytometric analysis revealed a small but distinct population of cells expressing IFN-β-tGFP in presence of the perturbator NS-1 (upper right quadrants in dot plots of Figure 2A and C). This fraction was negligible when Dox was added more than 12 h before infection (data not shown). For NS3/4A expressing cells this fraction was also observed, but it was much smaller (<0.5%) in all tested conditions. This may relate to the observed higher inhibition efficiency of NS3/4A (cf. Figure 2B). We hypothesized that these cells respond to NDV infection by IFN-β-tGFP expression before NS1 can antagonise its induction. IFN-expressing cells with delayed IAP-mediated inhibition could represent a new dynamic intermediate state of potential physiological relevance. Therefore, we performed time-lapse microscopic analysis to detect timing and course of expression of the IAP and IFN-β-tGFP (Figure 3A and D). Importantly, this allowed single cell correlation between IFN-activation and Tet-induced gene expression. We wanted to know if the occurrence of IFN-β-tGFP/NS1 double positive cells was due to ineffective inhibition, or whether this represents an intermediate state of perturbation. Thus, we tracked expression levels of IFN-β and the IAP by fluorescence microscopy in live cells (Figure 3B and E). To maximally induce the inhibitors, saturating levels of doxycycline (2 μg/ml) were applied. Importantly, monitoring intensity courses of IFN-β-tGFP and HaloTag-bound TMR revealed cells in which IFN-β-tGFP intensity stops increasing upon activation of expression of the IAP (grey arrows in Figure 3B and E). Time points from which no further increase of the reporter could be observed were identified as either a peak, or plateau of the intensity profile.

For NS3/4A expressing cells, this was observed in particular when either IFN-β-tGFP signal rose prior to a detectable TMR signal or in close temporal proximity (Figure 3E and Supplementary Figure S3), thus supporting our hypothesis. This phenomenon was also observed when RIG-I was stimulated with synthetic dsRNA (poly I:C) (Figure 3D). Thus, we could exclude the possibility that a side effect of the NS3/4A protease could be responsible for this observation. To test for potential correlation of these events we plotted the onset time points of NS3/4A expression after NDV infection against the time point of no further increase for IFN-β-tGFP (Figure 3E and Supplementary Figure S3; the earliest detectable TMR fluorescence signal above background at 300 ms exposure is indicated by black arrows and boxes; grey arrows and boxes indicate time points of no further increase). Linear regression analysis suggested that the majority of single-cell events could be attributed to a post-induction halt of IFN-β-tGFP by the increase of antagonist concentration (*R* = 0.68) with good significance (*P* = 0.0002) (Figure 3F).

NS1 lacks an obvious relationship between onset time and IFN-β inhibition (data not shown). In contrast, an arbitrary threshold (1000 a.u. indicated by dotted line, Figure 3B) significantly correlated with halted IFN-β expression (Figure 3C; *R* = 0.72; *P* = 0.0012). This suggested that NS1 levels needed to breach a threshold in order to successfully antagonise IFN activation underpinning the lower molecular effectiveness of NS1 compared to NS3/4A (Figure 2B).

In summary, these results suggest a retroactive inhibition of IFN-β induction by NS1 and NS3/4A, a novel delayed inhibition mechanism that is used by these antagonists.

### Delayed induction of IAV NS1 affects NF-κB nuclear accumulation

**Figure 4.**
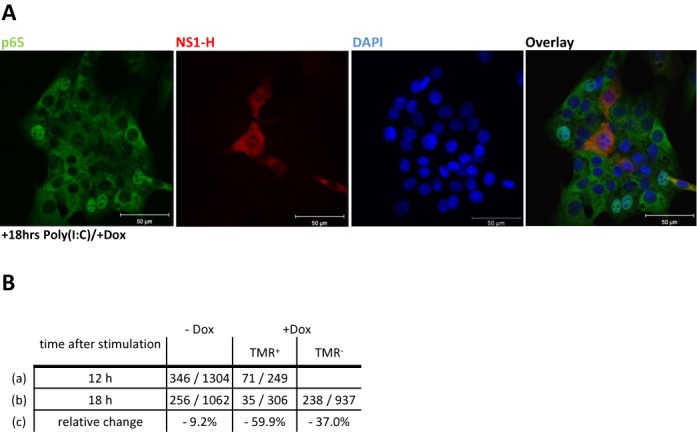
IAV NS1 expression decreases the period of NF-κB nuclear accumulation. NIH3T3 cells harbouring the Tet-inducible NS1 module were grown on glass coverslips and incubated with doxycycline (0.5 μg/ml) and TMR (100 nM) 2.15 h prior to poly I:C transfection. Cells were harvested 12 or 18 h after poly I:C stimulation, fixed, permeabilized and stained with primary rabbit NF-κB p65 followed by FITC labelled secondary anti-rabbit antibody (green). Cell nuclei were stained with DAPI (blue). TMR fluorescence (red) indicates NS1-H expression (**A**) Representative confocal microscopy images at 18 h after poly I:C stimulation; Scale bar: 50 μm. (**B**) The number and percentage of events showing p65 in the nucleus with or without TMR fluorescence was monitored at the indicated time points. The relative change (**c**) was calculated by dividing the fraction of cells with nuclear NF-κB at 12 h (**a**) by the difference of fractions at 12 h (a) and 18 h (**b**) after stimulation.

In order to identify the mechanism by which NS1 can stop IFN-β expression even after its activation, we analysed features of the upstream transcription factor NF-κB following IAP expression. NF-κB requires activation through the kinases IKKα/IKKβ/IKKγ (reviewed in (5)). We probed the NF-κB pathway downstream of RIG-I and analysed the nuclear accumulation of p65 in this reporter cell line harbouring the Tet-inducible NS1 module. We employed poly I:C lipofection to achieve better control of the RIG-I pathway response timing (6). Subsequent stimulation with doxycycline should result in a substantial fraction of cells showing the retroactive inhibition mechanism. Cells were induced with doxycycline, transfected with poly (I:C) and analysed for TMR fluorescence (NS1 expression) and p65 nuclear accumulation over a time period of 12 and 18 h. 18 h after poly I:C transfection, 256/1062 cells (24%) showed nuclear p65 in absence of doxycycline. In doxycycline-treated cells, we identified 238/937 (25%) TMR^−^ cells with p65 nuclear accumulation, however in the case of TMR^+^ expressing cells, only 35/306 (11%) exhibited nuclear translocation of p65 (Figure 4). This reduction of cells with p65 accumulation was consistent with a NS1-mediated inhibition of IFN induction (cf. Figure 2).

If expression of NS1 after activation of IFN gene transcription results in impairment of p65 translocation this might suggest that the TMR^+^ population should show decreased nuclear p65 over time. Therefore, we compared doxycycline-treated and untreated cells for alterations in the level of nuclear p65 at two critical time points. Cells that had not been treated with doxycycline showed a 9.2% decrease in p65 nuclear accumulation from 12–18 h after RIG-I stimulation. In contrast, when NS1 expression was induced by doxycycline, we observed a strong decrease of p65 nuclear accumulation in TMR^+^ cells (59.9%). This indicated that NS1 could indeed interfere with p65 nuclear translocation, even after the activation of IFN gene expression. Note that TMR^−^ cells also showed a decrease, but less pronounced, (Figure 4B). The latter is in agreement with the observation that a portion of the TMR^−^ cell population within the doxycycline treated culture showed NS1 activity even below the detection limit of TMR (cf. Figure 2).

To determine whether the duration of transcription factor activation downstream of RIG-I was generally decreased by IAV NS1, we measured the nuclear accumulation of IRF-7. A fusion of IRF-7 and eCFP was expressed in NIH3T3 cells together with the doxycycline-induced NS1 expression module, as shown in Figure 1A. Cells were stimulated with poly (I:C) and either treated with doxycycline or left untreated. Nuclear accumulation periods of IRF-7-eCFP were measured by time-lapse microscopy (Supplementary Figure S4). Interestingly, no significant variation could be observed between NS1 expressing and non-expressing cells, indicating that NS1 could not decrease the duration of IRF-7 nuclear accumulation. However, NF-κB inhibition by NS1 was sufficient to stop IFN-β expression, which is in agreement with the fact that both transcription factors are needed for transcriptional activation.

## DISCUSSION

Viral pathogens use a variety of different strategies to counteract the immune response. The IFN system is a major target of these self-protective mechanisms. IAPs encoded in virus nucleic acids are major determinants of pathogenicity and targets for antiviral therapy. However, little is known about their interaction with the dynamic IFN response. In particular, it has been unclear whether IAPs can have an effect at a stage after the initiation of IFN expression. This possibility is suggested by the current data and this supports the hypothesis that IFN activation via the RIG-I pathway can be reversed during the course of infection.

The analysis of the dynamic signalling processes following viral infection has been particularly hampered by the cell-to-cell heterogeneity of viral infection, the IFN response and other signalling processes. This requires the application of temporally resolved single cell analysis and non-invasive reporters that study the processes of interest (21). We have combined dual reporter live-cell imaging with a synthetic biology approach that allows us to interfere with dsRNA-activated IFN signalling and to elucidate part of the complex regulatory network that underlies this process (Figure 1A). This ensured controlled expression of IFN antagonistic proteins IAV NS1 and HCV NS3/4A independently of the virus’ life cycle. A classical doxycycline-dependent expression module was employed together with a complementary transcriptional repression by TetR-KRAB overcoming the limitations of basal transgene expression. While the TetOn system ensures precise adjustment of the level of transgene expression in a dose-dependent manner, a lack of temporal control was observed (Figure 1D and E) necessitating single-cell assays to study the dynamics of this host–pathogen interaction. Importantly, cell-to-cell heterogeneity is a fundamental aspect of viral infection and gene expression as demonstrated by various *in vitro* studies (22–24). This is considered to be even more pronounced *in vivo*, when the topological restrictions impair homogenous spread of virus through the tissue. The Tet system can mimic this natural stochasticity while allowing control over the population mean response time and extent of transgene expression.

Tet-controlled modules have been used extensively to control expression of transgenes in cells and in animals (25,26). This has provided information on the function of a plethora of genes. However, so far, the temporally resolved Tet-controlled gene expression within individual cells has not been described. Most, if not all, transcriptional systems have inherently stochastic components (27), while their functional outcome is widely unknown. By using live-cell imaging, we observed strong variability in the timing of the onset of doxycycline-dependent expression between individual cells. This heterogeneity was observed in multiple clones and was irrespective of the TetR-KRAB repressor protein (unpublished observation). As a consequence, single cells showed unique temporal patterns of doxycycline-induced IAP expression and also IFN-β expression shows stochastic temporal cell-to-cell variation (cf. ‘−Dox +NDV’ panels in Figure 2A and C; also described in (6)). Experimental limitations resulting from stochasticity of both systems were overcome in our approach by following individual cells via live-cell microscopy and correlating fluorescent signals showing doxycycline-induced IAP expression and cellular IFN-β promoter activity. We demonstrate that the correlation of single-cell responses to viral infection is very important to understand such dynamic processes in a stochastic environment.

In combination with the IFN-β-tGFP reporter, dose-dependent and time-dependent inhibition of the antiviral response was analysed in detail (Figure 2). Distinct efficacies and dynamics of NS1 and NS3/4A were elucidated, reflecting their mode of action. NS1 showed gradual inhibition dynamics with lower molecular efficiency compared to NS3/4A, while for NS3/4A a switch-like inhibition was observed. We found that IAV NS1 and HCV NS3/4A have hugely varying capacities for inhibition of the induction of IFN-β. While low concentrations of NS3/4A were sufficient to completely block activation of the IFN-β promoter, 40-fold higher levels of NS1 were required (Figure 2B). For NS3/4A, enzymatic cleavage of MAVS has been reported to block IFN activation, while for NS1 a repressive interaction with the TRIM25 ubiquitin ligase that activates RIG-I has been elucidated. In addition, other mechanisms have been hypothesized for these IAPs (28,29). These differential modes of blocking IFN activation might relate to the requirement for the virus to prevent IFN expression, or may relate to the differential establishment of either persistent infection (HCV) or acute infections (IAV).

We have uncovered a novel mechanism by which IAV NS1 and HCV NS3/4A are able to retroactively terminate IFN-β expression. We also show that NS1 induction specifically reduces the period of NF-κB nuclear accumulation (Figure 4B), leading to the observed early termination of IFN-β expression. Viral infection and host innate immune responses are dynamic processes that involve stochastic cell-to-cell variation. Thus, host–pathogen interactions at the cellular level cannot be strictly ordered temporal processes. Viral IAPs are often discussed as preventive factors inhibiting host immune responses. We suggest a dynamic model in which viruses have evolved to counteract the immune response at any stage, even when IFN activation has already started. We show that two viral IAPs of major clinical relevance, IAV NS1 and HCV NS3/4A, are capable of such late inhibition. NS1 specifically reduces the activity of NF-κB and this may well relate to its recently published inhibition of the IκB kinase (IKK) (30). In addition to its known interference with the RIG-I activating factor TRIM25, NS1 combines preventive and retroactive inhibition mechanisms.

Many viral proteins exhibit multiple diverse functions and also IAPs have been shown to use more than one antagonistic mechanism (7). These processes can block the IFN system at different stages using a variety of functional mechanisms. Dynamic cellular studies of these processes will give new insight into host–pathogen interaction. Moreover, it will also help to elucidate the mechanism of IAP inhibitors that are a major focus of current pharmaceutical research. Together, this has the potential to generate novel ideas for the antiviral therapies for the future.

In summary, the delayed inhibition of IFN expression by IAPs is a novel feature of viral antagonism and may represent a crucial mechanism in the race for supremacy between host and virus. Besides allowing comparative characterisation of viral IAPs, the cell culture system described here has the potential to serve for screening for compounds that selectively restore the immune response to viral infection.

## SUPPLEMENTARY DATA

Supplementary Data are available at NAR Online.

SUPPLEMENTARY DATA
