# Supplementary material for: Uncoupling of the dynamics of host–pathogen interaction uncovers new mechanisms of viral interferon antagonism at the single-cell level
Source: Nucleic Acids Res. 2014 Jun 4;42(13):e109. doi: 10.1093/nar/gku492 (PMC4117750; doi:10.1093/nar/gku492)
Supplement: SUPPLEMENTARY DATA [file supp_42_13_e109__index.html]

Uncoupling of the dynamics of host–pathogen interaction uncovers new mechanisms of viral interferon antagonism at the single-cell level — SUPPLEMENTARY DATA 

# Uncoupling of the dynamics of host–pathogen interaction uncovers new mechanisms of viral interferon antagonism at the single-cell level

## SUPPLEMENTARY DATA

**Files in this Data Supplement:**

- SUPPLEMENTARY DATA
