# Supplementary material for: Uncoupling of the dynamics of host–pathogen interaction uncovers new mechanisms of viral interferon antagonism at the single-cell level
Source: Nucleic Acids Res. 2014 Jun 4;42(13):e109. doi: 10.1093/nar/gku492 (PMC4117750; doi:10.1093/nar/gku492)
Supplement: SUPPLEMENTARY DATA [file supp_gku492_nar-00773-met-g-2014-File007.pptx]

## Slide 1
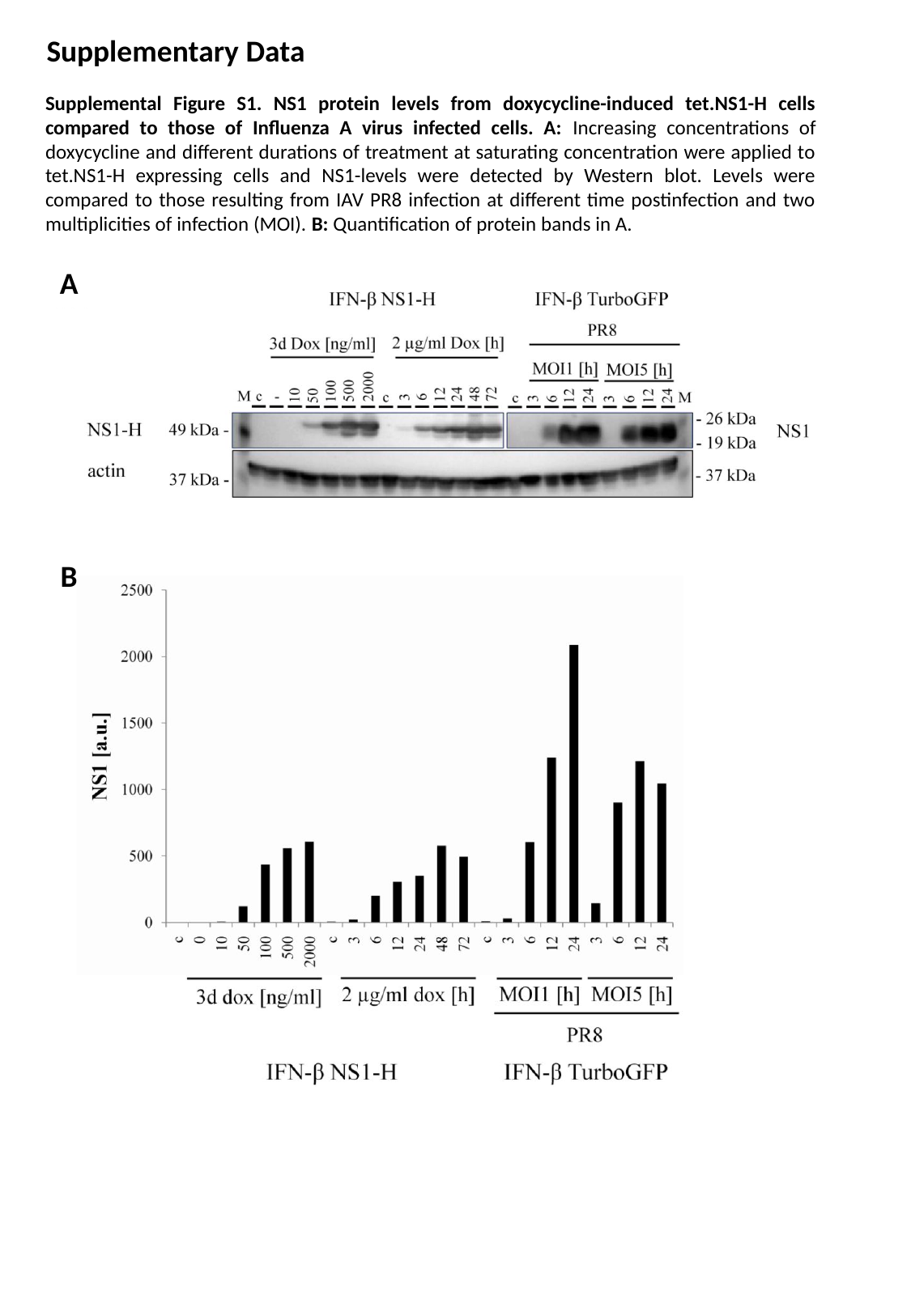

Supplementary Data
Supplemental Figure S1. NS1 protein levels from doxycycline-induced tet.NS1-H cells compared to those of Influenza A virus infected cells. A: Increasing concentrations of doxycycline and different durations of treatment at saturating concentration were applied to tet.NS1-H expressing cells and NS1-levels were detected by Western blot. Levels were compared to those resulting from IAV PR8 infection at different time postinfection and two multiplicities of infection (MOI). B: Quantification of protein bands in A.
A
B

## Slide 2
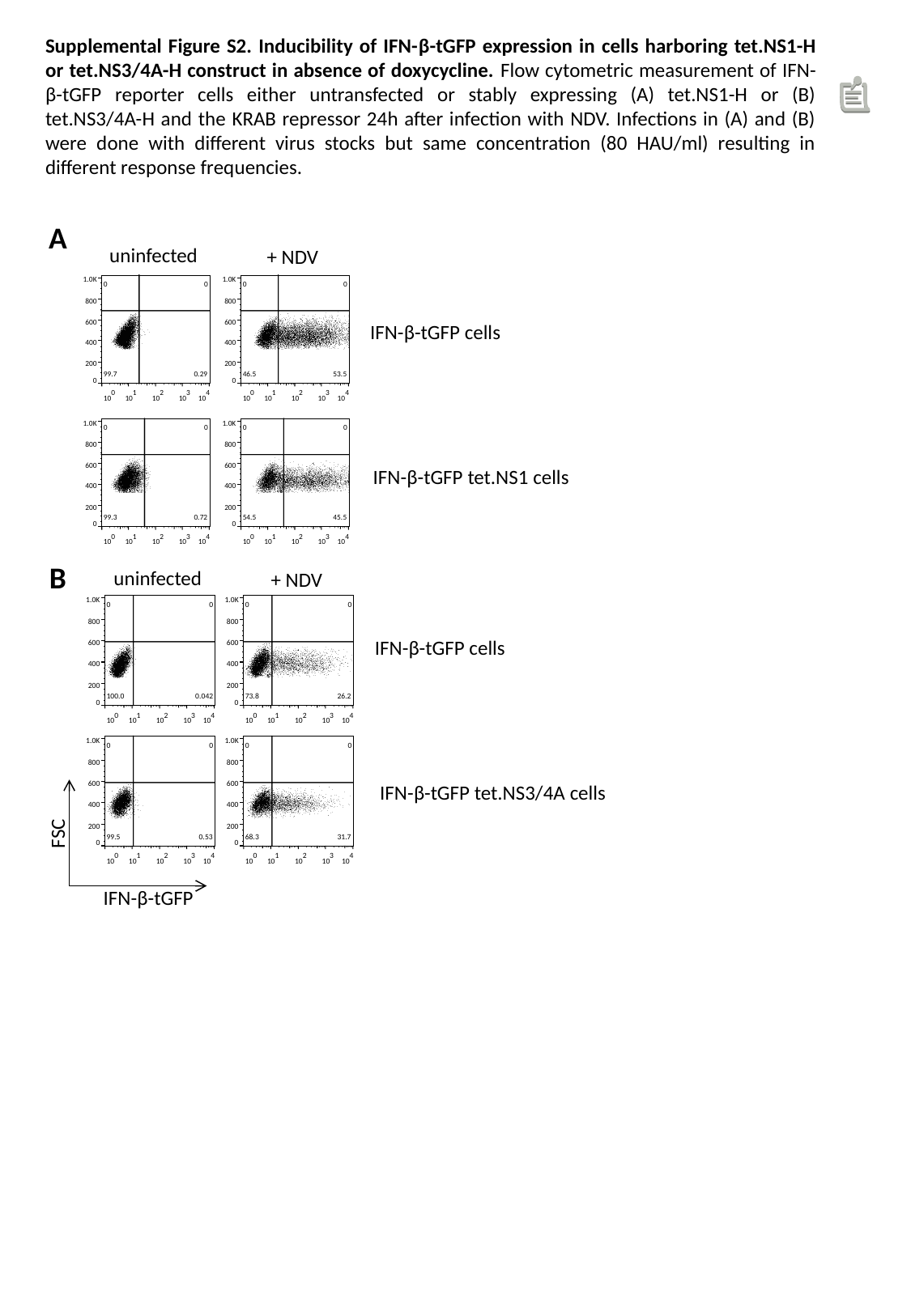

Supplemental Figure S2. Inducibility of IFN-β-tGFP expression in cells harboring tet.NS1-H or tet.NS3/4A-H construct in absence of doxycycline. Flow cytometric measurement of IFN-β-tGFP reporter cells either untransfected or stably expressing (A) tet.NS1-H or (B) tet.NS3/4A-H and the KRAB repressor 24h after infection with NDV. Infections in (A) and (B) were done with different virus stocks but same concentration (80 HAU/ml) resulting in different response frequencies.
A
uninfected
+ NDV
IFN-β-tGFP cells
IFN-β-tGFP tet.NS1 cells
B
uninfected
+ NDV
IFN-β-tGFP cells
IFN-β-tGFP tet.NS3/4A cells
FSC
IFN-β-tGFP

## Slide 3
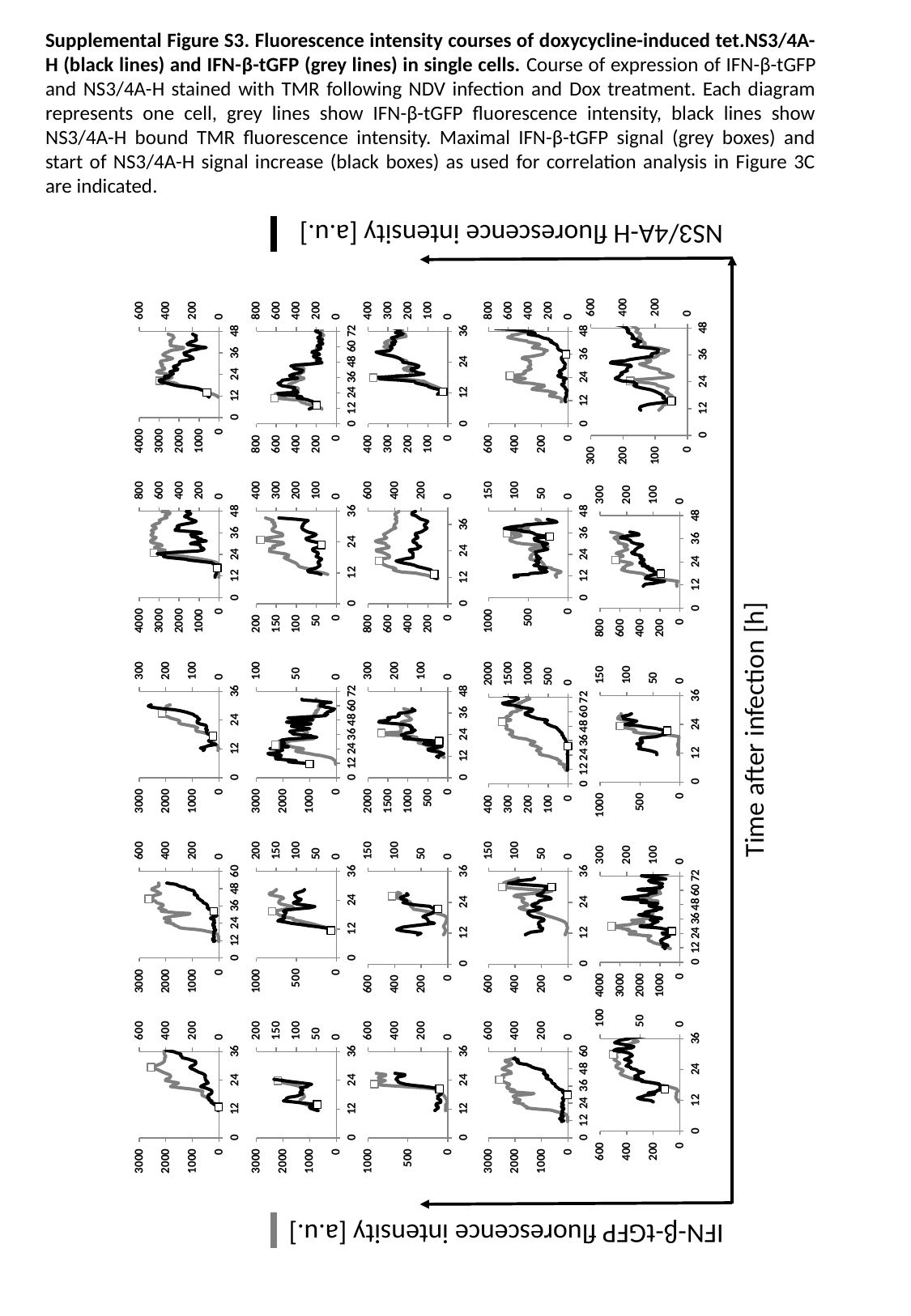

Supplemental Figure S3. Fluorescence intensity courses of doxycycline-induced tet.NS3/4A-H (black lines) and IFN-β-tGFP (grey lines) in single cells. Course of expression of IFN-β-tGFP and NS3/4A-H stained with TMR following NDV infection and Dox treatment. Each diagram represents one cell, grey lines show IFN-β-tGFP fluorescence intensity, black lines show NS3/4A-H bound TMR fluorescence intensity. Maximal IFN-β-tGFP signal (grey boxes) and start of NS3/4A-H signal increase (black boxes) as used for correlation analysis in Figure 3C are indicated.

## Slide 4
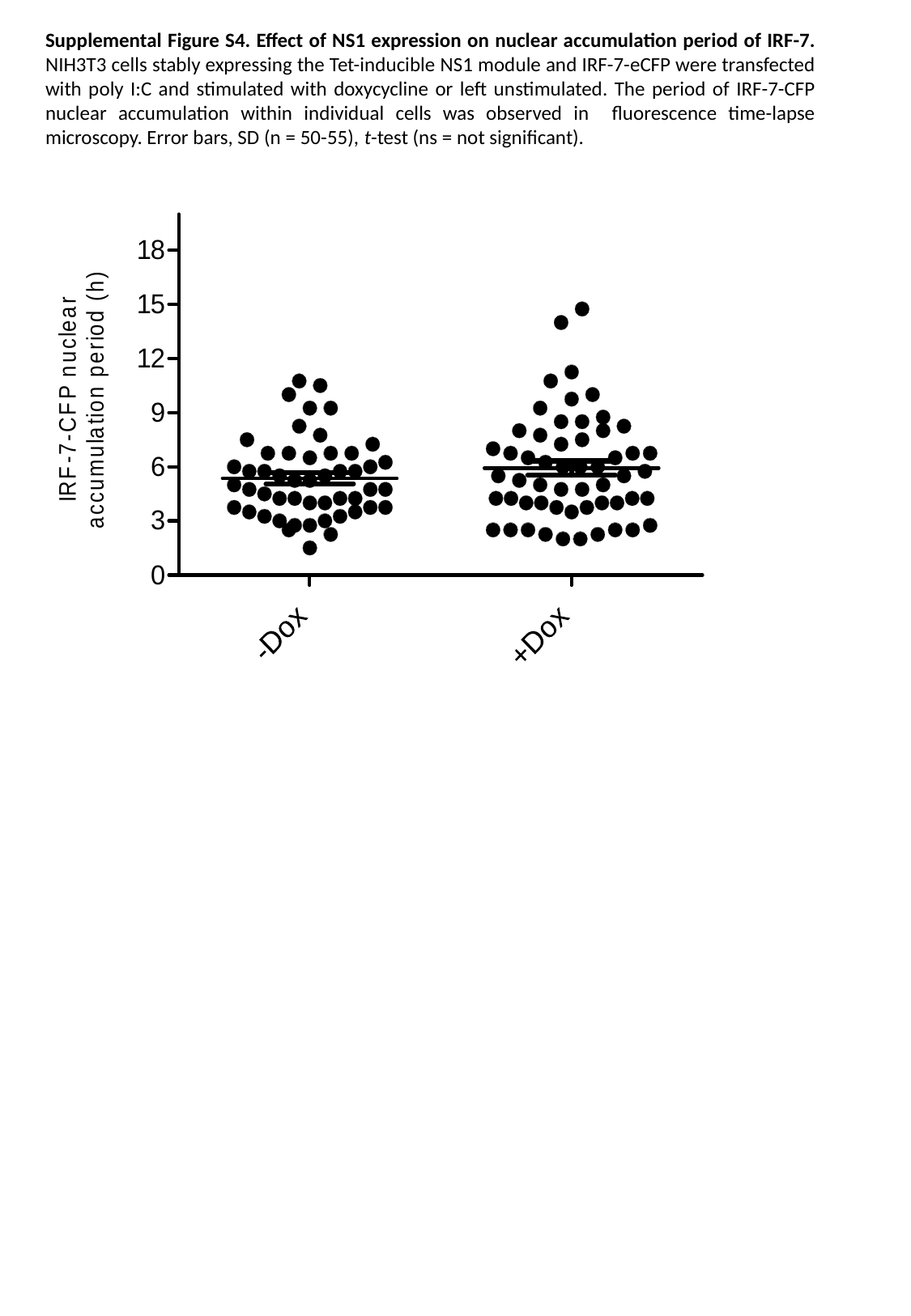

Supplemental Figure S4. Effect of NS1 expression on nuclear accumulation period of IRF-7. NIH3T3 cells stably expressing the Tet-inducible NS1 module and IRF-7-eCFP were transfected with poly I:C and stimulated with doxycycline or left unstimulated. The period of IRF-7-CFP nuclear accumulation within individual cells was observed in fluorescence time-lapse microscopy. Error bars, SD (n = 50-55), t-test (ns = not significant).
